# 

**Published:** 1914-07-18

**Authors:** 


### Medical Appointments.

Mr. E. F. Skinner, M.A., M.B., B.C., M.R.C.P., has been appointed to the post of tutor in clinical medicine at the University of Sheffield.

Mr. Gerald E. Stanley, M.S., F.R.C.S., has been appointed assistant surgeon at the Dreadnought Hospital of the Seamen's Hospital Society.

Mr. A. J. Scott Pinchin, M.D.(Lond.), M.R.C.P. (Eng.), has been appointed physician to out-patients at the Hampstead General and North-West London Hospital.

Dr. W. Mair has been appointed by the Metropolitan Asylums Board research pathologist. The laboratory accommodation for the research pathologist was hired from the Lister Institute of Preventive Medicine at a rental of £100 per annum, and the board has decided to renew the accommodation on the existing terms.

Mr. William Bainbridge, M.B., Ch.B. Edin., D.P.H. Bristol (house surgeon, Central London Ophthalmic Hospital), has been appointed honorary assistant ophthalmic surgeon to the Hull Royal Infirmary, in place of Dr. D. Matheson Mackay, M.D., C.M. Edin., M.R.C.S., L.R.C.P. Lond., who has succeeded Mr. A. Legge Roe as honorary ophthalmic surgeon.

Dr. P. L. J. Watkin, fourth assistant medical officer at Lambeth Infirmary, has been promoted to the office of third assistant medical officer, at a salary of £160 a year, rising by annual increments of £20 to a maximum of £200, in succession to Dr. J. A. Edmond, who has resigned. As the board has received no applications for the post of sixth assistant medical officer, Dr. N. L. M. Reader, M.B., B.Sc., has been engaged as locum tenens, at a salary of six guineas a week.

Mr. George Riddoch, M.B., Ch.B., has been appointed house physician to the West End Hospital for Diseases of the Nervous System. Mr. Riddoch graduated at Aberdeen University with first-class honours, gaining the John Murray Medal and Scholarship in 1913. He also gained the Fife Jamieson Medal in anatomy. He has since acted as junior demonstrator of anatomy in the University. At the British Medical Association meeting in July he is reading a paper on the "Sympathetic System of a Full-time Fœtus."

Owing to the recent appointment by the Metropolitan Asylums Board of a whole-time dentist for certain institutions, the appointment of Mr. E. Keen, who had been dentist to the training ship *Exmouth* since 1893, has been terminated, and he has applied to the board for a superannuation allowance or for compensation under the provisions of the Poor-Law Officers' Superannuation Act, 1896. The board cannot grant him a superannuation allowance, but, in the special circumstances, have decided to grant him a gratuity of £200 as compensation for loss of office.

Rates of Subscription: Twelve months, 6/6; Six months, 4/-; Three months, 2/-, post free to any address in the United Kingdom. Abroad, Twelve months, 8/8; Six months, 5/-; Three months, 2/6, post free.—Address The Subscription Dept., "The Hospital," The Hospital Building, 28/29 Southampton Street Strand, London W.C.

